# Using meta-analysis of Phase II trials to predict Phase III trial results

**DOI:** 10.1186/1745-6215-14-S1-O118

**Published:** 2013-11-29

**Authors:** Danielle Burke, Lucinda Billingham, Alan Girling, Richard Riley

**Affiliations:** 1 MRC Midland Hub for Trials Methodology Research, Birmingham, UK; 2 University of Birmingham, Birmingham, UK

## Objectives

Pharmaceutical companies use Phase II trial results to make decisions about proceeding to Phase III. We will show how a meta-analysis of results from multiple Phase II trials is informative toward this decision.

## Methods

We consider a meta-analysis of nine randomised Phase II trials comparing the efficacy of two therapies for acute myocardial infarction. Results for four outcomes were collected: intracranial haemorrhage, stroke, reinfarction and total mortality.

We apply univariate and multivariate random-effects meta-analysis methods, and use the obtained summary results to derive 95% prediction intervals, which give the predicted treatment effects for the four outcomes in a future trial. The multivariate approach jointly synthesizes all outcomes whilst accounting for their correlation. The methods are applicable in both frequentist and Bayesian frameworks. Predictions calculated are compared to results from subsequent Phase III trials.

## Results

The meta-analyses of Phase II trials show that the new treatment is promising for most outcomes. For example, the probability that the odds of stroke will be reduced by >10% in a future trial is 0.67. Importantly, the prediction intervals include the treatment effects that were seen in subsequent Phase III trials. These Phase III results have previously been described as 'contradictory' to the Phase II results, but our prediction intervals reveal this is not the case.

## Conclusions

The potential results of a Phase III trial can be informed by 95% prediction intervals derived from a Phase II meta-analysis. Such predictions could help pharmaceutical companies and funding bodies to prioritise interventions for Phase III evaluation.

